# Faecal pharmacokinetics of orally administered vancomycin in patients with suspected *Clostridium difficile *infection

**DOI:** 10.1186/1471-2334-10-363

**Published:** 2010-12-30

**Authors:** Milagros Gonzales, Jacques Pepin, Eric H Frost, Julie C Carrier, Stephanie Sirard, Louis-Charles Fortier, Louis Valiquette

**Affiliations:** 1 Department of Microbiology and Infectious Diseases, Université de Sherbrooke, 3001 12ème Avenue Nord, Sherbrooke, Quebec, Canada, J1 H 5N4; 2 Department of Medicine, Université de Sherbrooke, 3001 12ème Avenue Nord, Sherbrooke, Quebec, Canada, J1 H 5N4

## Abstract

**Background:**

Oral vancomycin (125 mg qid) is recommended as treatment of severe *Clostridium difficile *infection (CDI). Higher doses (250 or 500 mg qid) are sometimes recommended for patients with very severe CDI, without supporting clinical evidence. We wished to determine to what extent faecal levels of vancomycin vary according to diarrhoea severity and dosage, and whether it is rational to administer high-dose vancomycin to selected patients.

**Methods:**

We recruited hospitalized adults suspected to have CDI for whom oral vancomycin (125, 250 or 500 mg qid) had been initiated. Faeces were collected up to 3 times/day and levels were measured with the AxSYM fluorescence polarization immunoassay.

**Results:**

Fifteen patients (9 with confirmed CDI) were treated with oral vancomycin. Patients with ≥4 stools daily presented lower faecal vancomycin levels than those with a lower frequency. Higher doses of oral vancomycin (250 mg or 500 mg qid) led to consistently higher faecal levels (> 2000 mg/L), which were 3 orders of magnitude higher than the MIC_90 _of vancomycin against *C. difficile*. One patient receiving 125 mg qid had levels below 50 mg/L during the first day of treatment.

**Conclusions:**

Faecal levels of vancomycin are proportional to the dosage administered and, even in patients with increased stool frequency, much higher than the MIC_90_. Patients given the standard 125 mg qid dosage might have low faecal levels during the first day of treatment. A loading dose of 250 mg or 500 mg qid during the first 24-48 hours followed by the standard dosage should be evaluated in larger studies, since it might be less disruptive to the colonic flora and save unnecessary costs.

## Background

*Clostridium difficile *infection (CDI) is the main cause of nosocomial diarrhoea in industrialized countries. Recently, an increase in CDI incidence was observed in North America and Europe due to the emergence of an apparent toxin hyper-producing strain (NAP1/BI/027) [1-6]. For many years, metronidazole was preferred to vancomycin as the first-line treatment of CDI because of its lower cost, lower selection pressure for the emergence of vancomycin-resistant pathogens, and because it was considered as effective as vancomycin for most patients [7-12]. Oral vancomycin was recommended only for the most severe cases and for those relapsing after a course of metronidazole, based on its extremely high faecal levels, which are several hundred times higher than the MIC_90 _of vancomycin against *C. difficile*. Recently, this paradigm has been altered by reports of high rates of treatment failures and recurrences with metronidazole treatment and by randomized trials showing a potential advantage of oral vancomycin for severe cases [13,14].

The optimal dosage of oral vancomycin has not been well delineated. An underpowered study compared two different dosages (125 vs. 500 mg qid) without showing an impact on clinical outcomes [15]. Nevertheless, the Infectious Diseases Society of America (IDSA) recommends giving the highest dosage (500 mg qid) to patients with ileus, megacolon or hypotension [16]. The European Society of Clinical Microbiology and Infectious Diseases recommends giving the standard vancomycin dosage (125 mg qid) when oral therapy is possible for severe CDI but giving 500 mg qid via a nasogastric tube when oral therapy is impossible [17]. It would be very difficult to test such a strategy in clinical trials. However, pharmacokinetic studies of faecal vancomycin levels (as a proxy for colonic levels) can shed light on this debate. Early pharmacokinetic studies used bioassays in which dilutions of faecal material were incubated with an assay organism (*Bacillus subtilis*) of known sensitivity [18-20]. Subsequent work used a fluorescence polarization immunoassay [21,22] but only one documented sequential measurements in a few patients [21]. Some used Vancocin^® ^capsules, while many hospitals now administer orally generic IV vancomycin. To determine whether faecal levels of vancomycin vary according to stool frequency and dosage administered, we conducted a pharmacokinetic study of generic IV vancomycin administered orally to patients with suspected CDI.

## Methods

### Study design

A prospective observational study was conducted at the *Centre Hospitalier Universitaire de Sherbrooke *(CHUS), a 686-bed tertiary-care centre, from January to November 2009. Inpatients were invited to participate if (1) they were ≥ 18 years of age; (2) they had experienced three or more loose stools within the previous 24 hours; (3) they were already receiving oral vancomycin (using the IV formulation) for a suspicion of CDI, as prescribed by their attending physician. Patients unable to consent and/or who received vancomycin for less than 48 hours were excluded.

The institutional review board of the CHUS had approved our study protocol. Consenting patients were followed as long as they were receiving oral vancomycin or up to hospital discharge, whichever occurred first. Stool samples were collected up to three times per day for the first three days and daily for the remaining 11 days, and stored at -80°C until testing. Blood samples were drawn on days 1 and 3 to perform vancomycin serum levels. Patients were stratified in two groups according to stool frequency during the initial 72 hours of the treatment: ≥4 or ≤3 stools per day (this cut-off corresponded to the median value). Stool frequency was obtained from intestinal habits forms completed prospectively by patients' nurses as well as from nurses' notes in medical records. Patients were considered positive for CDI if they had either (1) a positive result with the cytotoxicity assay; (2) *C. difficile *detected by toxigenic culture; or (3) pseudomembranes evidenced by endoscopy. The standard vancomycin dosage referred to an intake of 125 mg qid. The other two regimens (250 and 500 mg qid) were considered higher doses. When two episodes of CDI occurred in the same patient more than 3 months apart, they were considered distinct events; an episode occurring within 3 months of a prior one was considered a relapse.

### Vancomycin assay

Faecal samples were diluted 1:40 in saline as to obtain values within the linear portion of the AxSYM assay (1.0 to 100 mg/L) and to reduce the impact of faecal material on the assay. This was further diminished by centrifuging 1 ml aliquots of the solution at 17,000 *g *for 10 minutes. The supernatant was then filtered through a 0.45 μm filter and sent to the clinical laboratory where vancomycin levels were determined using a fluorescence polarization assay on the AxSYM (Abbott, Abbott Park, Illinois). The AxSYM assay was also used to measure serum levels, as recommended by the manufacturer.

### Microbiological analyses

The first faecal sample was homogenized in BHI broth and spores were selected after killing all vegetative cells by mixing the faecal suspension with an equal volume of ethanol (50% final concentration) and incubating at room temperature for 45 minutes. The treated specimens were inoculated onto *C. difficile *moxalactam norfloxacin selective agar plates (CDMN, Oxoid, Nepean, Canada) supplemented with 5% sheep blood, 1 mM glycine and 0.1% taurocholic acid and were incubated anaerobically at 37°C for 24-48 hours. *C. difficile *identification was confirmed by colony morphology, distinct odour, and by a specific PCR reaction targeting the triose phosphate isomerase (*tpi*) gene [23].

### Strain typing

PCR ribotyping was performed using primers described previously with some modifications [24,25]. DNA fragments were separated through a 5% polyacrylamide gel and visualized under UV light after ethidium bromide staining. Profiles were analyzed with GelCompar II (Applied Maths) software.

### Data analysis

Data were analyzed using SAS, version 9.1.3 (Cary, North Carolina). Medians and ranges were reported in figures. Median faecal concentrations of vancomycin were compared using the Mann-Whitney test. Proportions were compared with the Pearson x^2 ^test or the Fisher exact test when numbers were small.

## Results

A total of 19 patients were enrolled in the study. Four patients were subsequently excluded: two because they received vancomycin for only one day after recruitment, and two others who had less than 2 stool specimens collected during their hospital stay. The final study sample included 15 patients (nine women and six men) with a median age of 63 (range: 43-99) years. Oral vancomycin was prescribed every six hours in all patients. Initial dosages (as selected by the attending physicians) were: 125 mg (n = 9), 250 mg (n = 4), and 500 mg (n = 2). Five patients had their dosage modified during their hospital stay. No patient experienced a CDI relapse within 90 days of enrolment.

Nine patients had a confirmed CDI (three had a positive cytotoxicity assay, four had evidence of pseudomembranous colitis, and two had both) while in the other six the diagnosis could not be confirmed. Toxigenic cultures were positive only in patients in whom the diagnosis had been proven by other methods. Based on PCR ribotyping, none of the cultured strains corresponded to the hypervirulent strain (ribotype 027). Of these nine patients, six had a peak leukocyte count greater than 15 × 10^9^/L, including three who were admitted to the ICU for severe CDI, and one who developed acute renal failure. Five were aged 60 years or more.

### Faecal levels

**Figure 1:**
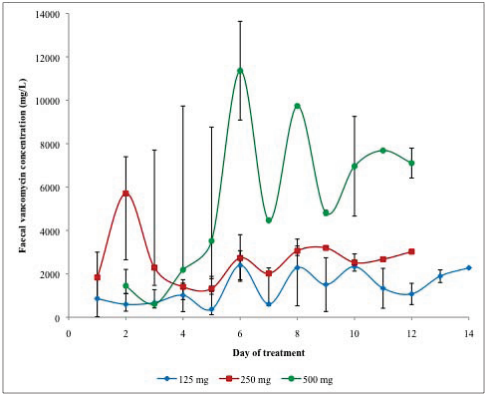
**Median faecal vancomycin levels achieved with different oral vancomycin dosages**. * Bars represent range. When no range is indicated, a single specimen was analyzed.

We compared faecal levels of vancomycin in patients with or without CDI confirmation, and found no statistically significant differences at any day of therapy (data not shown). Additionally, baseline data were similar in both groups (Table 1). Therefore, the rest of the analyses included all 15 patients, regardless of their CDI diagnosis. Day-to-day median faecal levels of vancomycin and ranges achieved according to dosage are illustrated in Figure 1. There were substantial variations between individual patients. Higher doses of oral vancomycin (250 mg or 500 mg qid) led to consistently higher faecal levels than the standard dose of 125 mg qid (Figure 1). Such differences were statistically significant from day 2 to day 4 inclusively. With the 250 mg qid dosage, faecal levels were generally above 2000 mg/L while with the 500 mg qid dosage levels were generally above 4000 mg/L. With the two higher dosages, faecal levels were always above 500 mg/L, even during the first few days of therapy, while with the 125 mg qid dosage some patients initially had levels below this threshold, and one had levels at 15 and 33 mg/L on day 1.

**Table 1:** Baseline characteristics in patients with and without confirmed CDI diagnosis.

	N (%) or median (IQR)	
**Variable**	**CDI-positive** **(n =9)**	**CDI-negative** **(n = 6)**

Age (years)	62 (50-86)	64 (59-65)

Peak creatinine (μmol/L)		
< 100	8 (89)	6 (100)
100-199	0	0
≥200	1 (11)	0

Peak leukocyte count × 10^9^/L < 10.0	0	1 (17)
10.0 - 19.9	2 (22)	2 (22)
≥20.0	7 (78)	3 (50)

Number of stools/day	4 (0-6)	5 (3-6)

Fever (first 48 hours)	3 (33)	5 (83)

Ileus	1 (11)	0

Initially prescribed dosage		
125 mg	5 (56)	4 (67)
250 mg	3 (33)	1 (17)
500 mg	1 (11)	1 (17)

Days of treatment	6 (5-15)	5 (4-10)

**Figure 2:**
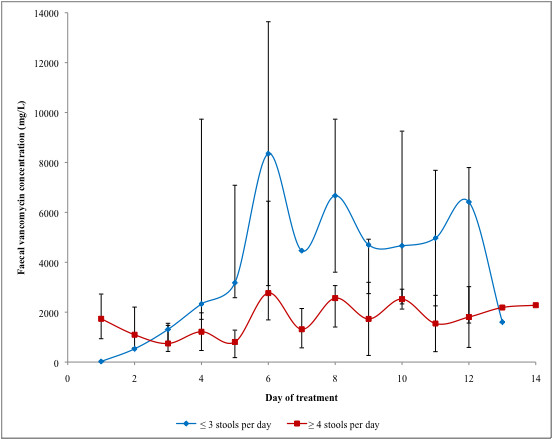
**Median faecal vancomycin concentrations achieved in patients stratified according to their stool frequency**. * Bars represent range. When no range is indicated, a single specimen was analyzed.

As shown in Figure 2, patients with higher (≥4 per day) stool frequency had lower vancomycin faecal levels, and the difference was statistically significant on days 4 and 5. These two groups did not differ with regard to the dosage and treatment duration used by their attending physicians: of 267 doses administered to patients with ≥4 stools per day, 123 (43%) were 250 mg or 500 mg while of 187 doses administered to patients with ≤3 stools per day, 91 (49%) were 250 mg or 500 mg] (p = 0.4). Average duration of treatment was 7 days (SD ± 4.1) for patient with ≥4 stools per day and 10.4 days (SD ± 4.5) in patients with ≤3 stools per day (p = 0.15). The patient with levels below 50 mg/L on day 1 was categorized as having mild diarrhoea but was not in ileus.

Three patients had their faecal levels measured after treatment completion. One had been in ileus and still had high levels (1422 mg/L) 48 hours after oral vancomycin was stopped. Two other patients had low levels on the day after their treatment was discontinued (23 and 38 mg/L).

### Serum levels

Serum samples were collected on day 1 and 3 after recruitment in 11 patients (two did not consent to provide a blood sample and two others received IV vancomycin for some other reason and were excluded). Serum concentrations ranged from 0 to 0.77 mg/L. Patients receiving higher dosage and/or with renal failure did not exhibit higher serum levels (data not shown).

## Discussion

Vancomycin has long been used only as a second-line treatment of CDI due to its high cost, if administered as the Vancocin^® ^capsule. Many hospitals now reduce their acquisition costs by administering orally the generic formulation of intravenous vancomycin, which is 10-fold cheaper. Our results support this approach and document that the faecal levels obtained with the IV formulation are as high as those measured in patients who had received the Vancocin^® ^capsule in previous studies [20,21].

As could have been predicted from the product's limited absorption, faecal levels of vancomycin increased in proportion with the dosage administered. For most of the duration of therapy, faecal levels achieved with the higher dosage are very unlikely to be more effective than those obtained with the standard 125 mg qid dosage, which themselves were often 500-1000 times higher than the MIC_90 _(1.0 mg/L) of vancomycin against *C. difficile *[26-29].

The faecal levels of vancomycin were influenced by stool frequency. Even if they were administered similar dosages of vancomycin, patients with ≥ 4 stools per day had lower levels compared to their counterparts, but the former's faecal levels were nevertheless consistently 100 to 1000 fold greater than the MIC_90_. Such findings are consistent with a higher output of loose stools resulting in some dilution of the vancomycin, but this would probably not be clinically relevant.

One of our patients receiving 125 mg qid had low faecal levels (only 15 and 33 mg/L) on day 1, as did some patients in a previous study [21]. Therefore it seems to us, based on our data and published literature, that for most CDI patients the only rationale for giving a higher dosage would be as a loading dose (250 or 500 mg qid) during the first 24-48 hours, especially in patients with some ileus for whom the delivery of orally administered vancomycin might be delayed. Beyond this initial period, there is little rationale for giving more than 125 mg qid, unless the patient has very severe ileus: neither our study nor previous work specifically addressed this subpopulation of CDI cases. However, before modifying clinical practices, additional studies are required to evaluate lower dosages, in the hope that they would be as effective against *C. difficile *while being less destructive for the normal colonic flora. Indeed, faecal levels of vancomycin with the 125 mg qid dosage are often higher than the MIC_90 _of this drug against *Bacteroides *and *Prevotella *spp (64-256 mg/L) and consequently might alter the normal colonic flora and favour relapses [30-32].

In patients receiving orally administered vancomycin, serum levels were always below 1 mg/L, even in patients given the highest 500 mg qid dosage and in those with renal failure. These low serum levels are irrelevant with regard to potential nephrotoxicity or ototoxicity. A handful of case reports have demonstrated considerable systemic absorption (within the 5-10 mg/L range) after oral administration of vancomycin in patients who had either severe renal disease or were receiving high doses of oral vancomycin (250 or 500 mg qid) [33-36]. Only one published case reported a patient with significant systemic absorption and normal renal function but the patient suffered from severe graft-versus-host disease of the gastrointestinal tract [33]. Our study is the largest to quantify the systemic absorption of oral vancomycin. Our data suggest there is no need to monitor vancomycin serum levels in patients receiving oral vancomycin for CDI, unless they receive high doses for prolonged periods with ileus and/or end-stage renal failure, as stated in the IDSA guidelines [16].

Our study had limitations. Its small sample size limited its power to detect statistically significant differences between subgroups. Some of our patients were eventually proven not to have CDI. Since its main focus was on vancomycin pharmacokinetics, we elected to include all patients at the onset of their vancomycin prescription in order to have the most samples possible rather than wait for CDI confirmation. In the absence of universally accepted criteria, we chose stool frequency to evaluate the impact of such a "diluting factor" on the faecal vancomycin levels but admittedly this is an imperfect measure of diarrhoea severity.

## Conclusions

Faecal levels of vancomycin are roughly proportional to the dosage administered and even in patients with high stool frequency are consistently 100 to 1000 times higher than the MIC_90_. Patients given the standard 125 mg qid dosage might have low faecal levels during the first 24 hours of treatment. A loading dose of 250 mg or 500 mg qid during the first 24-48 hours followed by the standard dosage should be evaluated in larger pharmacokinetics studies, since it might be less disruptive to the colonic flora and save unnecessary costs.

## Competing interests

LV has been on the speakers' bureau for Wyeth, has served on advisory boards for Oryx Pharmaceuticals, Abbott and Wyeth, and has received compensation to conduct clinical trials with Genzyme, Wyeth, Merck, Optimer, BD and Arpida. JP has been on the speakers' bureau for Wyeth and Merck, and has served on advisory boards for Bayer, Wyeth, Viropharma and Acambis. MG, SS, EHF, JCC, and LCF have no competing interest to report.

## Authors' contributions

MG carried out the faecal assays and participated in the design of the study and coordination, performed the statistical analysis and helped to draft the manuscript. EHF performed faecal assay's validation. SS and LCF carried out the molecular analysis. LV and JP conceived the study, performed the statistical analysis and helped to draft the manuscript. All authors read and approved the final manuscript.

## Pre-publication history

The pre-publication history for this paper can be accessed here:

http://www.biomedcentral.com/1471-2334/10/363/prepub
